# Cardiac Autonomic Modulation in Seizure Disorders With and Without Interictal Epileptiform Discharges: A Retrospective Heart Rate Variability Analysis Using Archived Digital EEG Recordings

**DOI:** 10.7759/cureus.112765

**Published:** 2026-07-16

**Authors:** Raihan Mannan, Md. Zabihullah, Kamlesh Jha, Saran AK, Zakiyyah Tasneem

**Affiliations:** 1 Physiology, All India Institute of Medical Sciences, Patna, IND

**Keywords:** cardiac autonomic function, electroencephalography, heart rate variability, interictal epileptiform discharges, seizure

## Abstract

Background

Cardiac autonomic dysfunction is increasingly recognized in epilepsy and has been implicated in cardiovascular complications and sudden unexpected death in epilepsy (SUDEP). Heart rate variability (HRV) provides a non-invasive measure of cardiac autonomic modulation; however, the influence of interictal epileptiform discharges (IEDs) on resting HRV remains uncertain. This study compared HRV among patients with seizure disorders with and without IEDs and a non-seizure neurological comparison group using ECG signals extracted from archived routine electroencephalography (EEG) recordings.

Methods

This retrospective observational study included 45 adults (15 per group): seizure disorder with IEDs, seizure disorder without IEDs, and non-seizure neurological complaints. A stable, artifact-free five-minute lead II ECG segment was extracted from archived digital EEG recordings after completion of activation procedures. Short-term HRV analysis was performed using LabChart Pro (ADInstruments, Dunedin, New Zealand) and included time-domain, frequency-domain, and nonlinear parameters. Groups were compared using one-way ANOVA or the Kruskal-Wallis test, with appropriate post hoc analyses.

Results

Demographic characteristics were comparable across the three groups. pNN50 differed significantly among the groups (F(2,42) = 5.127, p = 0.010), with higher values in the non-seizure group than in both seizure groups. Total power (F(2,42) = 3.353, p = 0.045) and high-frequency (HF) power (F(2,42) = 3.905, p = 0.028) also differed significantly, with post hoc differences observed between the non-seizure comparison group and the seizure without IED and seizure with IED groups, respectively. No significant differences were observed in LF power, LF/HF ratio, or nonlinear HRV indices. No HRV parameter differed significantly between the two seizure groups.

Conclusions

Patients with seizure disorders demonstrated lower vagally mediated HRV than the non-seizure neurological comparison group, whereas the presence of IEDs was not associated with measurable additional alterations in resting cardiac autonomic modulation.

## Introduction

Epilepsy is one of the most common chronic neurological disorders, affecting approximately 50 million people worldwide and representing a major cause of neurological disability across all age groups [1]. Although recurrent unprovoked seizures are the hallmark of epilepsy, increasing evidence suggests that epilepsy is a network disorder involving widespread cortical and subcortical dysfunction extending beyond seizure generation [2]. Among the various extra-neurological manifestations of epilepsy, autonomic nervous system dysfunction has received considerable attention because of its association with cardiovascular instability, arrhythmias, and sudden unexpected death in epilepsy (SUDEP) [3,4].

The brain and cardiovascular system are closely interconnected through the central autonomic network, which includes the insular cortex, amygdala, anterior cingulate cortex, hypothalamus, and brainstem autonomic nuclei [3]. Epileptic activity involving these structures may disrupt autonomic regulation by altering sympathetic and parasympathetic balance, leading to abnormalities in heart rate, blood pressure, and cardiac rhythm [5]. Chronic autonomic dysfunction has been implicated as one of the important mechanisms underlying increased cardiovascular morbidity and mortality among patients with epilepsy [3].

Heart rate variability (HRV) is a simple, non-invasive, and reproducible method for assessing cardiac autonomic modulation by quantifying beat-to-beat variations in sinus rhythm [6]. Time-domain, frequency-domain, and nonlinear HRV indices provide complementary information regarding parasympathetic activity, sympathetic modulation, sympathovagal balance, and overall autonomic adaptability [7]. Reduced HRV, particularly reductions in vagally mediated indices such as root mean square of successive differences (RMSSD), HF power, and SD1, has been associated with impaired autonomic regulation in epilepsy [3,8]. However, relatively few studies have examined whether the presence of interictal epileptiform discharges (IEDs) is associated with additional impairment of resting cardiac autonomic function.

Interictal epileptiform discharges, including spikes, sharp waves, and spike-and-wave complexes, reflect abnormal neuronal synchronization and persistent cortical excitability even during the seizure-free period [9]. Persistent activation of cortical regions involved in the central autonomic network may contribute to altered cardiac autonomic regulation during the interictal state [3]. Therefore, patients demonstrating IEDs on routine EEG may exhibit greater autonomic dysfunction than patients with seizure disorders without demonstrable epileptiform activity [10,11]. Clarifying this relationship may improve our understanding of brain-heart interactions in epilepsy and the potential utility of HRV as a marker of autonomic dysfunction.

In India, nearly 12-13 million people are estimated to be living with epilepsy, accounting for a substantial proportion of the global epilepsy burden [12]. Bihar, one of the most populous states in India, has a predominantly rural population with limited access to specialized neurological services in many districts. Consequently, tertiary referral centers receive a large number of patients with suspected seizure disorders requiring electroencephalographic evaluation. Routine digital EEG recordings obtained in such centers frequently include a simultaneously acquired ECG channel, providing an opportunity to retrospectively evaluate cardiac autonomic function without additional physiological recordings. Although archived EEG-derived ECG recordings may be subject to variability in recording conditions and signal quality, careful selection of standardized, artifact-free ECG segments may enable reliable short-term HRV assessment. This approach may expand the utility of existing neurophysiology databases for retrospective autonomic research.

Therefore, the present study aimed to compare cardiac autonomic modulation among patients with seizure disorders and interictal epileptiform discharges, patients with seizure disorders without interictal epileptiform discharges, and patients with non-seizure neurological complaints using HRV analysis derived from ECG signals extracted from archived digital EEG recordings. We further explored whether the presence of interictal epileptiform discharges was associated with additional alterations in resting cardiac autonomic modulation among patients with seizure disorders.

## Materials and methods

Study design and setting

This retrospective observational study was conducted in the Central Neurophysiology Laboratory, Department of Physiology, All India Institute of Medical Sciences (AIIMS), Patna, India. Archived digital electroencephalography (EEG) recordings performed over a six-month period were retrospectively screened for eligibility. EEG studies had been requested by various clinical departments, including Neurology, General Medicine, Psychiatry, Neurosurgery, Pediatrics, Emergency Medicine, and other specialties, for evaluation of suspected neurological disorders. Patient identifiers were removed before analysis to ensure confidentiality.

Study population

Participants were classified based on clinical diagnosis and EEG findings into three groups.

Group I (Seizure Disorder With Interictal Epileptiform Discharges)

Group I consists of patients with clinically diagnosed seizure disorder demonstrating IEDs, including spikes, sharp waves, spike-and-wave complexes, or polyspikes on routine EEG.

Group II (Seizure Disorder Without Interictal Epileptiform Discharges)

Group II consists of patients with clinically diagnosed seizure disorder whose routine EEG did not demonstrate interictal epileptiform discharges.

Group III (Non-seizure Comparison Group)

Group III consists of patients referred for routine EEG because of non-seizure neurological complaints such as headache, dizziness, vertigo, syncope, and altered sensorium. These patients had no documented history of seizure disorder, and their EEG recordings showed no evidence of epileptiform abnormalities.

**Figure 1:**
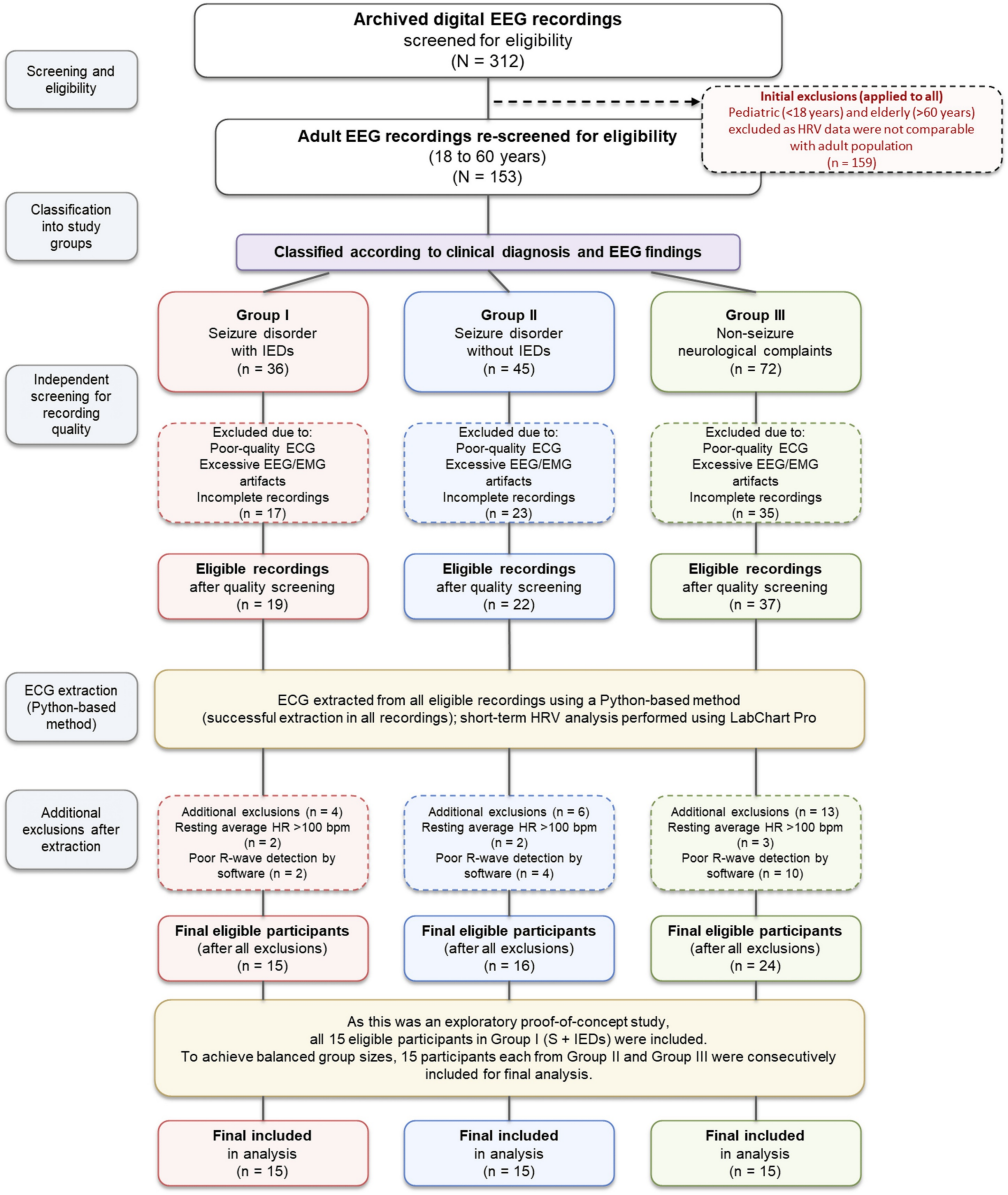
Flow diagram of participant screening and selection.

Archived digital EEG recordings obtained during the study period were initially screened for eligibility (N = 312). Pediatric (<18 years) and elderly (>60 years) patients were excluded because HRV parameters are not directly comparable with the adult population. The remaining adult EEG recordings (N = 153) were then classified according to clinical diagnosis and EEG findings into Group I (seizure disorder with IEDs, n = 36), Group II (seizure disorder without IEDs, n = 45), and Group III (non-seizure neurological complaints, n = 72). Each group was independently screened for recording quality by two investigators experienced in clinical neurophysiology, and disagreements were resolved by consensus. Recordings with poor-quality ECG, excessive EEG/EMG artifacts, or incomplete data were excluded, resulting in the exclusion of 17, 23, and 35 recordings from Groups I, II, and III, respectively. ECG was successfully extracted from all remaining eligible recordings using a Python-based method, and short-term HRV analysis was performed using LabChart Pro (ADInstruments, Dunedin, New Zealand). Additional exclusions were then made for resting average heart rate > 100 bpm and poor R-wave detection by the software, resulting in final eligible samples of 15, 16, and 24 participants in Groups I, II, and III, respectively. As this was an exploratory proof-of-concept study, all 15 eligible participants in Group I were included, and 15 participants each from Groups II and III were consecutively included to achieve balanced group sizes for comparison (Figure 1).

EEG acquisition

Routine EEG recordings were performed using the RMS EEG-32 SuperSpec Digital EEG System (Medicare Systems Pvt. Ltd., Chandigarh, India). Nineteen scalp electrodes were positioned according to the International 10-20 System. Electrode impedance was maintained below 10 kΩ before recording. EEG signals were acquired at a sampling frequency of 256 Hz using a band-pass filter of 1-70 Hz with a 50-Hz notch filter to reduce power-line interference. Standard EEG activation procedures, including hyperventilation and intermittent photic stimulation, were performed whenever clinically indicated. A lead II ECG was recorded simultaneously throughout the EEG examination.

ECG extraction and signal processing

Native EEG recordings were exported in European Data Format (EDF) for offline processing. A custom Python-based pipeline was developed to extract the simultaneously recorded ECG channel from each EDF file. The extracted ECG signal was converted into a tab-delimited (.txt) format compatible with LabChart Pro Version 8. This workflow enabled retrospective HRV analysis from archived EEG recordings without requiring separate ECG acquisition. The ECG extraction pipeline was validated by visual comparison of extracted ECG traces with the original recordings before HRV analysis.

ECG segment selection

For each participant, a continuous five-minute ECG segment was manually selected after completion of the routine EEG activation procedures (hyperventilation and intermittent photic stimulation) to minimize the transient autonomic effects of these activation procedures. The selected segment represented a stable resting-state recording with normal sinus rhythm and was free from movement, muscle, and electrode artifacts, ectopic beats, and interictal epileptiform discharges on the simultaneously recorded EEG. Only high-quality ECG segments with a minimum duration of five minutes were considered eligible for HRV analysis.

HRV analysis

HRV analysis was performed using the HRV Module of LabChart Pro Version 8. R-wave detection was performed automatically and manually verified. Beat detection errors and artifacts were corrected before analysis according to standard recommendations of the Task Force of the European Society of Cardiology and the North American Society of Pacing and Electrophysiology [6].

The analyzed HRV parameters included time-domain indices (average heart rate, average RR interval, median RR interval, SDRR/SDNN (standard deviation of RR/NN intervals), standard deviation of successive differences (SDSD), root mean square of successive differences (RMSSD), and pNN50)), frequency-domain indices (total power, low-frequency (LF) power, high-frequency (HF) power, LF and HF expressed in normalized units, and the LF/HF ratio), and nonlinear indices (SD1, SD2, and the SD1/SD2 ratio).

Statistical analysis

Microsoft Excel (Microsoft Corp., Redmond, WA, USA) and IBM SPSS Statistics for Windows, Version 24.0 (Released Year; IBM Corp., Armonk, NY, USA) were used for data entry and statistical analysis. Continuous variables were recorded as mean ± SD or median with IQR, depending on the normality of the data. Frequency and percentage were used to express categorical variables. Normality was assessed using the Shapiro-Wilk test and visual inspection of histograms and Q-Q plots, while homogeneity of variances was evaluated using Levene’s test. Normally distributed variables with homogeneous variances were compared using one-way ANOVA, followed by Tukey’s honestly significant difference (HSD) test for post hoc pairwise comparisons when the overall ANOVA was significant. Variables that did not satisfy the assumption of normality were analyzed using the Kruskal-Wallis test. Categorical variables were compared using Pearson’s chi-square test. Effect sizes are reported as eta-squared (η²) for one-way ANOVA and epsilon-squared (ε²) for the Kruskal-Wallis test. A two-sided p-value < 0.05 was considered statistically significant.

## Results

A total of 45 participants were included in the study, with 15 participants in each group. The mean age was comparable among the three groups (29.87 ± 10.90; 31.20 ± 12.38; and 29.20 ± 11.73 years, respectively; p = 0.893). The overall cohort comprised 22 males and 23 females. Sex distribution was similar across the three groups (χ² = 2.846, df = 2, p = 0.241), indicating that the groups were comparable with respect to demographic characteristics (Table 1).

**Table 1:** Demographic and clinical characteristics of the study participants. Age and baseline average heart rate were compared using one-way ANOVA, whereas sex distribution was compared using the Pearson chi-square test.

Variable	Group I	Group II	Group III	p-value
Number of participants	15	15	15	-
Age (years), mean ± SD	29.87 ± 10.9	31.20 ± 12.38	29.20 ± 11.73	0.893
Male, n (%)	6 (40)	10 (66.67)	6 (40)	
Female, n (%)	9 (60)	5 (33.33)	9 (60)	
Mean heart rate (bpm), mean ± SD	77.06 ± 10.85	77.82 ± 11.64	72.28 ± 8.26	0.294
Primary clinical indication	Seizure	Seizure	Non-seizure	-
EEG findings, n (%)	IED present	IED absent	IED absent	-
	Spike, 5 (33.3)			
	Sharp wave, 9 (60)			
	Polyspike, 1 (6.7)			

Among the time-domain HRV parameters, only pNN50 differed significantly among the three groups (one-way ANOVA, F = 5.127, p = 0.010; η² = 0.196). Tukey's post hoc analysis demonstrated significantly higher pNN50 values in the non-seizure comparison group than in both the seizure with IED group (p = 0.017) and the seizure without IED group (p = 0.030).

Although SDNN, SDSD, RMSSD, and SD1 did not reach statistical significance (p > 0.05), all demonstrated moderate effect sizes (η² ≈ 0.11), suggesting a trend toward reduced vagally mediated cardiac autonomic modulation in patients with seizure disorders (Table 2).

**Table 2:** Comparison of time-domain HRV parameters. Data are presented as mean ± SD. One-way ANOVA was used for normally distributed variables. Effect sizes are reported as η² (ANOVA). HRV: heart rate variability, SDNN: standard deviation of NN intervals, SDSD: standard deviation of successive differences, RMSSD: root mean square of successive differences.

Time-domain HRV parameters	Group I	Group II	Group III	p-value	Effect size
SDNN (ms)	42.8 ± 13.62	42.77 ± 8.5	50.69 ± 9.88	0.083	0.112
SDSD (ms)	37.37 ± 15.15	40.62 ± 13.24	50.72 ± 20.09	0.079	0.114
RMSSD (ms)	37.33 ± 15.11	40.55 ± 13.2	50.64 ± 20.05	0.079	0.114
pNN50 (%)	12.45 ± 3.14	13.77 ± 2.44	28.74 ± 5.66	0.010	0.196

Among the frequency-domain parameters, total power differed significantly among the study groups (one-way ANOVA, F = 3.353, p = 0.045; η² = 0.138). Tukey’s HSD post hoc analysis demonstrated significantly higher total power in the non-seizure comparison group than in the seizure without IED group (adjusted p = 0.044).

HF power also differed significantly among the three groups (one-way ANOVA, F = 3.905, p = 0.028; η² = 0.157). Tukey’s HSD post hoc analysis demonstrated significantly higher HF power in the non-seizure comparison group than in the seizure with IED group (adjusted p = 0.035).

LF power (Kruskal-Wallis test, H = 3.439, p = 0.179; ε² = 0.034), LF/HF ratio (Kruskal-Wallis test, H = 0.386, p = 0.825; ε² < 0.001), LF power (normalized units), HF power (normalized units), SD2, and SD1/SD2 ratio did not differ significantly among the groups (Table 3).

**Table 3:** Comparison of frequency-domain and non-linear HRV parameters. Data are presented as mean ± SD or median (IQR), as appropriate. One-way ANOVA was used for normally distributed variables, whereas variables marked with "#" were analyzed using the Kruskal-Wallis test. Effect sizes are reported as η² (ANOVA) and ε² (Kruskal-Wallis). HRV: heart rate variability, LF: low frequency, HF: high frequency.

Frequency domain and non-linear HRV parameters	Group I	Group II	Group III	p-value	Effect size
Total power (ms²)	1940.37 ± 1194.87	1711.68 ± 609.17	2660.6 ± 1221.73	0.045	0.138
^#^LF power (ms²)	416.87 (235.05)	438.35 (242.83)	722.55 (548.05)	0.179	0.034
LF power (nu)	48.2 ± 22.8	43.25 ± 13.1	42.76 ± 20.21	0.692	0.017
HF power (ms²)	549.65 ± 405.6	629.23 ± 412.17	1115.71 ± 864.76	0.028	0.157
HF power (nu)	48.13 ± 19.78	55.2 ± 11.39	54.76 ± 20.49	0.479	0.034
^#^LF/HF	1.52 (1.4)	0.87 (0.51)	1.02 (0.73)	0.825	0.00
SD1 (ms)	26.43 ± 10.71	28.72 ± 9.37	35.87 ± 14.2	0.079	0.114
SD2 (ms)	53.8 ± 18.23	52.91 ± 9.6	61.02 ± 11.51	0.214	0.071
SD1/SD2	0.50 ± 0.19	0.54 ± 0.13	0.59 ± 0.22	0.421	0.040

Overall, no significant differences were observed between the seizure with IED and seizure without IED groups for any time-domain, frequency-domain, or nonlinear HRV parameter. Thus, within the limitations of this exploratory study, the presence of IEDs was not associated with measurable additional alterations in resting cardiac autonomic modulation.

Distributions and post hoc pairwise comparisons of significant HRV parameters

**Figure 2:**
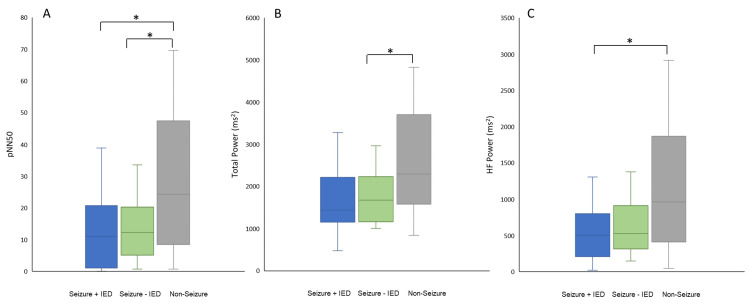
Boxplot showing the distributions and Tukey’s HSD post hoc pairwise comparisons of (A) pNN50 (%), (B) total power (ms²), and (C) HF power (ms²) across the study groups. Brackets indicate the compared groups, and asterisks (*) denote statistically significant pairwise differences (p < 0.05).

Figure 2 illustrates the distributions of pNN50, total power, and HF power across the three study groups, with significant pairwise differences identified using Tukey’s HSD post hoc test indicated by brackets and asterisks. The non-seizure comparison group showed higher values for these parameters, while considerable overlap was observed between the seizure with IED and seizure without IED groups.

## Discussion

Patients with seizure disorders demonstrated lower vagally mediated HRV than the non-seizure comparison group. Specifically, the non-seizure group had significantly higher pNN50 than both seizure groups, higher HF power than the seizure with IED group, and higher total power than the seizure without IED group. These findings suggest altered cardiac parasympathetic modulation in patients with seizure disorders and are consistent with previous studies reporting reductions in HF power, RMSSD, and pNN50 in epilepsy [13-15].

Among HRV indices, pNN50 and HF power predominantly reflect cardiac vagal modulation [6]. The higher values observed in the non-seizure group therefore indicate greater parasympathetic modulation, whereas the lower values in the seizure groups are consistent with reduced vagal influence. This pattern aligns with previous studies demonstrating reduced interictal HRV in patients with epilepsy [10,11,15]. However, no significant differences were observed between patients with and without IEDs, suggesting that the presence of IEDs on routine EEG was not associated with measurable additional alterations in resting cardiac autonomic modulation in this cohort.

Several mechanisms may explain the similar HRV profiles observed in the two seizure groups. Recurrent seizures and long-standing epileptic activity may produce cumulative alterations within the central autonomic network, including the insula, amygdala, hypothalamus, and brainstem autonomic pathways, potentially impairing cardiac vagal regulation [3,13,14]. Autonomic function may also be influenced by epilepsy duration, seizure frequency, epilepsy syndrome, disease severity, and antiseizure medication exposure [16]. Certain antiseizure medications, including carbamazepine, have been associated with alterations in HRV, although the available evidence is not entirely consistent [17]. As these clinical variables were not available in the present retrospective study, their potential contributions could not be evaluated. Thus, the observed autonomic alterations may reflect the broader effects of the underlying seizure disorder and its treatment rather than the presence of IEDs on a single routine EEG recording.

Reduced vagally mediated HRV and lower overall HRV have previously been associated with autonomic dysfunction in epilepsy and have been investigated as potential markers related to SUDEP risk [4,14]. However, the present study did not assess SUDEP, cardiovascular outcomes, or their temporal relationship with HRV. Therefore, no inference regarding risk prediction can be made from these findings. Larger prospective longitudinal studies are required to determine whether EEG-derived HRV measures have prognostic value for adverse cardiovascular outcomes or SUDEP.

A notable aspect of this exploratory study is the use of ECG signals extracted from archived routine EEG recordings for short-term HRV analysis. This approach demonstrates the feasibility of repurposing routinely acquired neurophysiological data for retrospective assessment of cardiac autonomic modulation without additional recordings or patient burden. However, further validation in larger prospective cohorts is required before its clinical utility can be established.

Limitations and future directions

The present study is limited by its retrospective design, small sample size, and single-center setting. Additionally, epilepsy-related detailed clinical information, including disease duration, seizure frequency, seizure burden, epilepsy syndrome, time since the last seizure, and the type and duration of antiseizure medication use, was not evaluated. Future prospective studies with larger cohorts, healthy controls, and longitudinal follow-up are required to validate these findings and determine their clinical significance.

## Conclusions

In conclusion, patients with seizure disorders demonstrated reduced vagally mediated HRV compared with the non-seizure neurological comparison group, while the presence of interictal epileptiform discharges was not associated with measurable additional autonomic impairment. HRV derived from routine EEG recordings may provide a feasible, non-invasive approach for assessing cardiac autonomic modulation in epilepsy.
